# Silent pheochromocytoma and paraganglioma: Systematic review and proposed definitions for standardized terminology

**DOI:** 10.3389/fendo.2022.1021420

**Published:** 2022-10-17

**Authors:** Georgiana Constantinescu, Cristina Preda, Victor Constantinescu, Timo Siepmann, Stefan R. Bornstein, Jacques W. M. Lenders, Graeme Eisenhofer, Christina Pamporaki

**Affiliations:** ^1^ Department of Endocrinology and Diabetes, University Hospital Carl Gustav Carus, Technische Universität Dresden, Dresden, Germany; ^2^ Department of Endocrinology, Grigore T. Popa University, Iasi, Romania; ^3^ Department of Health Care Sciences, Center for Clinical Research and Management Education, Dresden Inter-national University, Dresden, Germany; ^4^ Center of Clinical Neuroscience, University Clinic Carl-Gustav Carus, Dresden University of Technology, Dresden, Germany; ^5^ Department of Neurology, University Hospital Carl Gustav Carus, Technische Universität Dresden, Dresden, Germany; ^6^ Department of Health Care Sciences, Center for Clinical Research and Management Education, Dresden International University, Dresden, Germany; ^7^ Division of Diabetes & Nutritional Sciences, Faculty of Life Sciences & Medicine, King's College London, London, United Kingdom; ^8^ Department of Internal Medicine, Radboud University Medical Centre, Nijmegen, Netherlands; ^9^ Institute of Clinical Chemistry and Laboratory Medicine, University of Dresden, Dresden, Germany

**Keywords:** pheochromocytoma, paragangliomas, silent, clinically silent, biochemically negative

## Abstract

Pheochromocytomas and paragangliomas (PPGLs) are rare neuroendocrine tumors with heterogeneous clinical presentations and potential lethal outcomes. The diagnosis is based on clinical suspicion, biochemical testing, imaging and histopathological confirmation. Increasingly widespread use of imaging studies and surveillance of patients at risk of PPGL due to a hereditary background or a previous tumor is leading to the diagnosis of these tumors at an early stage. This has resulted in an increasing use of the term “silent” PPGL. This term and other variants are now commonly found in the literature without any clear or unified definition. Among the various terms, “clinically silent” is often used to describe the lack of signs and symptoms associated with catecholamine excess. Confusion arises when these and other terms are used to define the tumors according to their ability to synthesize and/or release catecholamines in relation to biochemical test results. In such cases the term “silent” and other variants are often inappropriately and misleadingly used. In the present analysis we provide an overview of the literature and propose standardized terminology in an attempt at harmonization to facilitate scientific communication.

## Introduction

Pheochromocytomas and paragangliomas (PPGLs) are neuroendocrine tumors derived from chromaffin cells of the adrenal medulla or extra-adrenal paraganglionic tissue. Clinical presentation of PPGL depends on capacity of the tumors to synthesize and release catecholamines to impact adrenergic receptors in multiple tissues and organs (1). Signs and symptoms vary accordingly and are highly heterogeneous. Biochemical diagnosis depends primarily on measurements of plasma or urinary metanephrines, the O-methylated metabolites of catecholamines (2).

The past several decades have seen increased use of the term “silent PPGL”, presumably reflecting increased discovery of tumors that do not produce the usual signs and symptoms of catecholamine excess consequent to their discovery as incidentalomas or during routine surveillance based on hereditary risk or a previous tumor. The term “silent PPGL” or other variants have become common in the literature without any clear or consistent link to the clinical and biochemical presentation of affected patients. In some cases, the term “silent” is used to describe the absence of signs and symptoms of catecholamine excess (3–5). In other cases, use of the terms “silent” and “non-functioning” tumors have been employed equivalently to describe patients with PPGL who present without signs and symptoms but in whom it is not always clear whether the tumors produce catecholamines (6–8). In other cases, the term “non-secretory” or “non-secreting” has been employed to designate patients with absence of secretory symptoms or lack of functional activity (9–11). Appropriate definitions according to the ability of the tumors to synthesize catecholamines (functional/non-functional), release catecholamines (secretory/non-secretory) or according to the presence of positive or negative biochemical test results (biochemically positive/negative) are essential for scientific communication.

The need for unified nomenclature to better describe “silent PPGLs” has become increasingly important given the widespread use of anatomic imaging and expansion of surveillance programs for patients at risk of PPGL due to genetic predisposition or a previous tumor (12, 13). The aim of the present analysis is first to review the relevant literature and then propose standardized terminology in an attempt to improve scientific communication about PPGLs according to their ability to synthesize, store, metabolize and secrete the catecholamines responsible for the heterogeneous clinical presentation of the tumors.

## Overview of the literature

**Figure 1:**
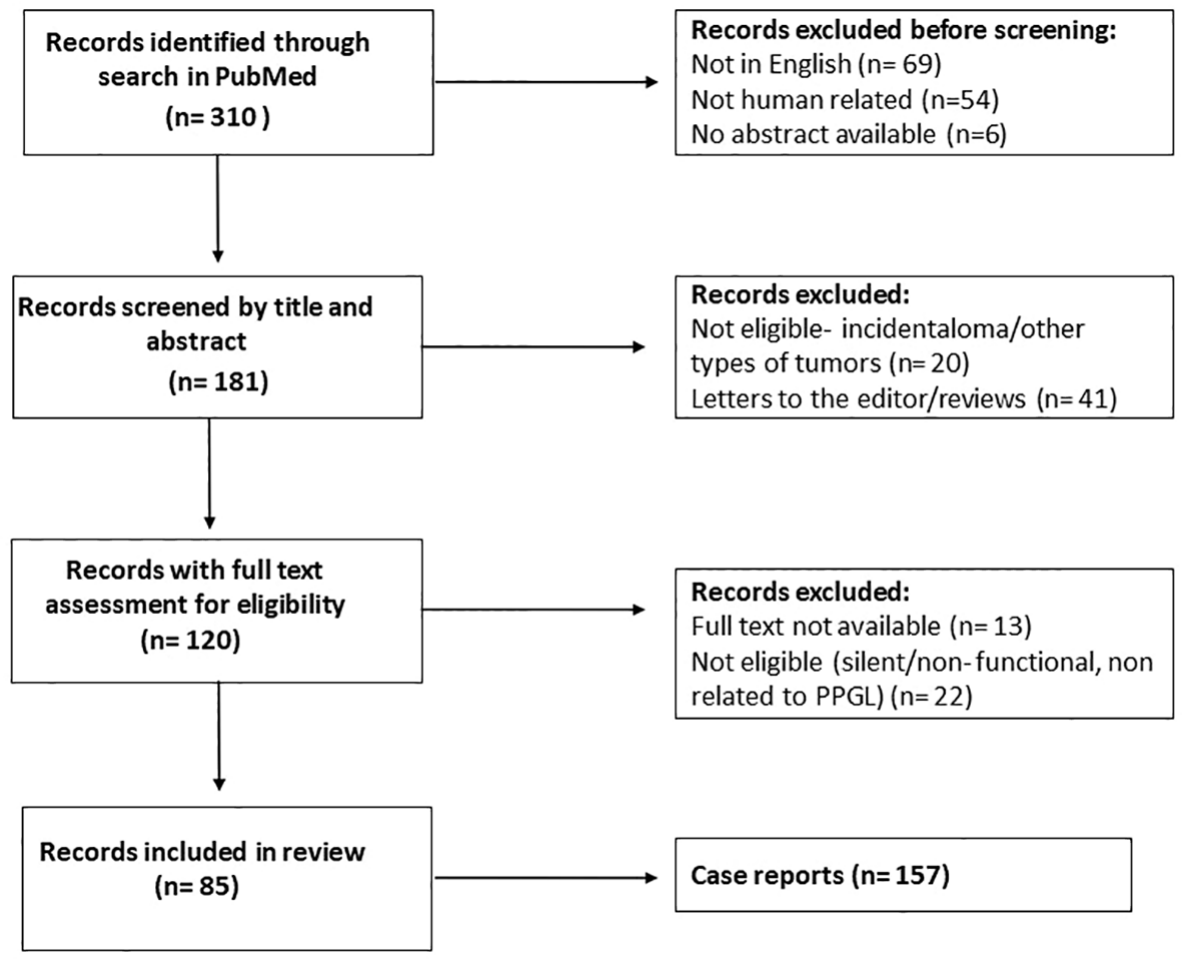
Flow chart after review of the literature. After screening and text analysis, 157 cases were included in the final analysis.

Two researchers (GC and VC) independently searched PubMed for articles published in English from 1-1-1980 to 30-08-2021. The following search terms were used: ((pheochromocytoma [MeSH Terms]) or (pheochromocytoma [Title/Abstract]) or (paraganglioma [Title/Abstract])) AND ((silent [Title/Abstract]) or (nonfunctioning [Title/Abstract])). Based on title and abstract, GC and VC independently selected the papers that reported on patients with PPGLs. Subsequently, full text articles were downloaded. Articles without extractable data of individual cases were excluded. GC and VC accessed all papers and extracted data. Items not explicitly reported were noted as ‘not mentioned’. Three hundred ten articles were initially identified through PubMed. One hundred twenty-nine articles were excluded for lack of eligibility after review of the title and abstract (not in English, not human related or no abstract available) (**Figure 1**). Screening by title and article excluded a further 61 articles, while screening after reading the full text reduced the eligible articles to 85, which covered a total of 157 cases in the final analysis. The PRISMA flow diagram is shown in **Figure 1**. Due to data heterogeneity no meta-analysis was carried out.

### Patient characteristics

Among the 157 cases reported in the final analysis (**Table 1**), 48% were females. Patients presented more often with extra-adrenal (62%) than adrenal tumors, whereas prevalence of metastatic disease was 25%. The most common reasons for biochemical testing were detection of incidental adrenal lesions (62%) due to abdominal or other non-specific complaints. Finally, in 36% of cases, diagnosis was established during surveillance and follow up. There remained only 3 cases (2%) where specific signs and symptoms of catecholamine excess provided the initial reason for the diagnostic work up.

**Table 1:** Patient characteristics.

Total Number	157
Female, n (%)*	58 (48%)
**Age at initial diagnosis, years (SD)**	45 (±14.7)
**Location**	
**Extra-adrenal**	62% (74/157)
Adrenal	38% (60/157)
Head and Neck	15% (25/157)
**Diagnostic setting**	
Incidentaloma	62% (97/157)
Abdominal complaints	48.3% (45/93)
Imaging performed for non-specific complaints	51.7% (48/93)
Surveillance/Follow up	36% (57/157)
Specific signs and symptoms	2% (3/157)
**Tumor composition** (reported in 29 cases)	
Solid	31% (9/29)
Cystic	31% (9/29)
Hemorrhagic and/or necrosis	38% (11/29)
**Maximal tumor diameter (cm)** (reported in 81 cases)	5.5 (0.25-25)**^#^**
**Metastatic disease** (reported in 68 cases)	25% (17/68)
**Plasma free metanephrines** (reported in 45 cases)	
Results within the normal range	89% (40/45)
Elevated results	11% (5/45)
**Urinary metanephrines** (reported in 57 cases)	
Results within the normal range	75.4% (43/57)
Elevated results	24.5% (14/57)

### Terminology according to clinical and biochemical phenotypes

In 56 patients (36%) the authors used the term ‘clinically silent’ (3–9, 14–57) to describe the absence of the “classic triad” and/or hypertension. In eight cases, although patients were defined to have ‘clinically silent’ tumors, they presented with symptoms that could have been related to catecholamine excess, such as sweating, weight loss, vomiting and nausea (3, 5, 9, 15, 24, 31, 48, 57). In addition to the term “clinically silent” to define the absence of signs and symptoms, other terms were used based on biochemistry. In particular, 55 cases were defined by the authors as “biochemically silent” (4, 11, 16, 18, 21, 22, 58–64), 59 as “non-functional” (6–8, 17, 19, 35–39, 41–50, 52–54, 65–89) and two as “non-secretory” PPGL (9, 10) and 7 patients presented “negative markers” (90).

Among 55 cases defined by the authors as “biochemically silent”, test results of plasma metanephrines with respective reference intervals were available in only 15 cases (11, 22, 58, 59, 64) (**Table 2**). Among those 15 cases, test results and reference intervals for plasma catecholamines and metanephrines were reported in five cases (11, 58) and for urinary metanephrines (+/- catecholamines) in 25 cases (16, 21, 22, 58, 59, 61, 63, 64). All plasma results showed values below upper cut-offs of stipulated reference intervals. Urinary metanephrines were below upper cut-offs of reference intervals in all except one case when at the 3 year follow up the patient presented with increased urinary normetanephrine (21). Finally, test results and reference intervals for plasma and urinary metanephrines were available in only eight cases (22, 58, 59, 64), whereas in only four patients test results and reference intervals were available for catecholamines and metanephrines in both plasma and urine (58). In all these patients the results showed normal values.

**Table 2:** Biochemical tests in patients according to authors’ classification of catecholamine biochemical activity.

						Non-functional/Non-secretory/			
		Biochemically silent				Negative markers			
		No	TR	RI	References^§^	No	TR	RI	References^§^
Plasma									
	Cat	1	1	1	16	9	2	1	87, 90^¥^
	Met	36	15	25	22, 59,61*, 64	14	10	10	65^¶^, 86, 89,90^¥^
	Cat& Met	5	5	5	11, 58	0	0	0	–
	CgA	4	3	3	16, 58	1	1	1	9
Urine									
	Cat	3	0	0	–	6	1	1	84
	Met	36	9	25	21^¶^, 22, 61*,64	16	16	14	9^¥^, 65^¶^, 79, 86, 87,90^¥^
	Cat& Met	15	15	15	16, 58, 59, 63	2	1	1	43
	VMA	2	0	0	-	9	3	3	36, 41, 88

No, total number of patients with; Cat, catecholamines; Met, metanephrines; CgA, chromogranin A; VMA, vanillylmandelic acid; TR, test result reported; RI, reference interval reported; *referred to RI but had not test results, ^§^ references of manuscripts presenting RI and TR.

^¶^ increased concentration, ^¥^ referred to TR but had no RI.

Similarly, among 59 patients classified by the authors with ‘non-functional’ tumors (6–8, 17, 19, 35–39, 41–50, 52–54, 65–89), test results and reference intervals for plasma metanephrines were only mentioned in ten cases (65, 86, 89) and for urinary metanephrines in fourteen (65, 79, 86, 87) (**Table 2**). In one patient referred to as having a non-functional tumor, both plasma and urinary measurements indicated increased concentrations of normetanephrine (65), while in another patient only plasma metanephrines were measured and found to be increased (19). Two patients were referred to as having ‘non-secretory’ PPGL (9, 10) while 7 patients presented with negative markers according to the authors (90).

The method of measurement for plasma and urinary catecholamines and metanephrines was mentioned only in eight studies, either as high performance liquid chromatography (9, 29, 58, 59, 63) or liquid chromatography with mass spectrometry (23, 61, 86).

## Proposed definitions for a standardized approach

Review of the literature revealed that the term “silent” was used in a highly variable fashion according to widely differing circumstances. The term “clinically silent” was mainly used to describe the absence of symptoms of catecholamine excess, which is appropriate. However, definitions according to the ability of tumors to synthesize and/or release catecholamines were inconsistently used according to biochemical test results. In some cases, biochemical test results were not even mentioned. In order to address these shortcomings, we propose use of standardized terminology that may be useful in an attempt for harmonized and more consistent descriptions of how patients may present with silent PPGL.

### Clinically silent PPGLs

“Clinically silent” PPGLs are more common than usually appreciated. Starting in the 1980s patients with pheochromocytoma who were both normotensive and asymptomatic began to be identified incidentally upon imaging studies for purposes other than suspicion of the tumor (91), a trend that has increased subsequently with the broadening use of imaging studies (92). Before this, the almost exclusive mode of discovery was based on clinical suspicion according to the presence of signs and symptoms (93).

Starting in the late 1980’s, with the advent of surveillance programs involving patients with von Hippel Lindau (VHL) syndrome or multiple endocrine neoplasia (MEN), it became apparent that most patients identified in this way also had clinically silent tumors (94–100). Discovery at an earlier stage by positive biochemical tests and/or imaging studies when tumors are small and secrete insufficient amounts of catecholamines to produce typical manifestations of the tumor provides the main explanation for such presentations. This underlies the likelihood that all PPGLs start out without eliciting signs and symptoms of catecholamine excess. Nevertheless, some PPGLs can be relatively large and/or secrete large amounts of catecholamines and still remain clinically silent, indicating that other factors can contribute to a normotensive and asymptomatic presentation (101–103).

Apart from tumor size and the extent of catecholamine secretion, other factors that may contribute to the absence of signs and symptoms in patients with PPGLs include the types of catecholamines secreted, the sustained or episodic nature of catecholamine secretion and adaptive physiological responses to catecholamine secretion. About half of all pheochromocytomas produce a combination of epinephrine and norepinephrine, while most others and particularly paragangliomas produce nearly exclusively norepinephrine (104). These differences depend on expression of phenylethanolamine-N-methyltransferase (PNMT), the enzyme that converts norepinephrine to epinephrine (105). Some tumors that show minimal expression or complete lack of dopamine-β-hydroxylase, the enzyme that converts dopamine to norepinephrine, may produce and secrete combinations of dopamine and norepinephrine or occasionally in some paragangliomas only dopamine.

Dopamine has negligible actions on α- and ß-adrenoreceptors and primarily elicits vasodepressor responses *via* actions mediated by an array of dopamine receptors particularly important in mesenteric and renal vascular beds (106, 107). This clarifies why patients with dopamine-producing paragangliomas may be asymptomatic and are usually normotensive or may even suffer from hypotension (105, 108). Epinephrine has variably more potent agonist actions on α- and ß-adrenoreceptors than norepinephrine (109). Epinephrine has particularly stronger actions than norepinephrine on ß_2_-adrenoreceptors responsible for vasodilation in skeletal muscle. According to studies involving intravenous (i.v.) infusions of epinephrine and norepinephrine in healthy subjects, increases in systolic blood pressure relative to the increased plasma catecholamines are larger for epinephrine than norepinephrine (110). On the other hand, diastolic blood pressure shows small decreases compared to increases with norepinephrine.

With the above factors in mind, the lower potency of norepinephrine than epinephrine on adrenoceptors may contribute to the higher proportion of normotensive and clinically silent norepinephrine-producing tumors in patients with VHL syndrome than those with epinephrine-producing tumors in MEN2 (95); however, there are other factors that can contribute to a clinically silent phenotype among patients with PPGLs.

Among various factors to be considered to account for clinically silent PPGL, it should not be overlooked that blood pressure and other responses associated with increased plasma concentrations of norepinephrine are much larger when due to increased secretion of norepinephrine from sympathetic nerves than associated with i.v. infusion of norepinephrine and therefore presumably also secretion of norepinephrine from a PPGL. For example, although a little more than 2-fold increase in plasma norepinephrine to 3.6 nmol/L during sympathetic activation results in a 25 mmHg increase in systolic blood pressure (111), the same increase in norepinephrine during its i.v. infusion results in only a 4 mmHg increase in systolic blood pressure, while a 25 mmHg increase in blood pressure requires circulating concentrations of norepinephrine of over 20 nmol/L (110). These differences reflect concentration gradients of the transmitter between sites of release at neuroeffector junctions in the adventitia of blood vessels compared to the bloodstream and differing geographic locations of adrenoceptors within blood vessels impacted by neuronal versus hormonal secretion (112). More than 80% of norepinephrine in the blood stream is derived from neuronal rather than hormonal sources and circulating norepinephrine is largely irrelevant as a hormone compared to epinephrine, which also targets different populations of adrenoceptors. The above considerations explain why increases in plasma norepinephrine resulting from tumoral secretion of the catecholamine may not evoke signs and symptoms of catecholamine secretion until increases are reasonably large.

In addition to the aforementioned factors, physiological adaptation can also contribute to a clinically silent presentation in the face of high circulating concentrations of catecholamines. This can take the form of both hypovolemia or a redistribution of blood volume as a compensatory response to increased blood pressure or diminished responsiveness of adrenoceptors to activation by catecholamines after prolonged adrenergic stimulation (101, 113). Of additional relevance are repeated observations that tumors that produce exclusively norepinephrine tend to secrete the catecholamine in a sustained manner whereas those that produce epinephrine tend to more often show an episodic pattern of catecholamine secretion (114–116). Sustained secretion of norepinephrine in the former noradrenergic tumors might be expected to contribute to tachyphylaxis more than in tumors that secrete catecholamines in widely spaced episodes.

It should also be appreciated that although noradrenergic tumors show a usually more sustained pattern of catecholamine secretion than adrenergic tumors, these tumors are also characterized by lower secretory stores of catecholamines (117); this might further impact the clinical presentation by limiting overall secretory capacity.

Apart from head and neck paragangliomas that usually do not produce appreciable catecholamines, relatively low tissue catecholamine stores are particularly common in patients with paragangliomas due to mutations of succinate dehydrogenase subunit B and D (*SDHB* and *SDHD*) genes (117). Tumors due to *SDHB* mutations show a particularly immature phenotype that often involves relatively high tissue contents of dopamine. In order to produce and secrete sufficient amounts of catecholamines to cause related signs and symptoms, these tumors often reach a large size before diagnosis, which may contribute to their predisposition to metastasize. Also, occasionally found are tumors that produce only dopamine (108) or those that do not produce any catecholamines and which remain clinically silent until they produce local mass effects.

Finally, and as will be covered in more detail later, although almost all PPGLs produce catecholamines, a significant proportion do not secrete catecholamines in amounts sufficient to produce diagnostically meaningful increases in plasma or urinary catecholamines or related signs and symptoms of catecholamine excess. These tumors typically can only be detected by measurements of metanephrines in urine or more ideally plasma. Most often these tumors are adrenergic in nature and may only become apparent clinically after catecholamine secretion is provoked.

Taking all the above into consideration, several factors may contribute to the absence of clinical manifestations of PPGLs including small tumor size and minimal catecholamine secretion, as well as the type and pattern of catecholamine secretion, adrenoceptor desensitization and other compensatory responses to the disease. However, in many cases patients may present with nonspecific signs and symptoms that are overlooked by clinicians, especially if there is coexistence of other clinically confusing conditions (e.g., diabetes, menopause, migraine). Based on our review of the literature, most clinicians still focus their interest on the presence or absence of hypertension (6, 8, 19, 21, 28, 30, 47, 56), although it has been repeatedly shown that this feature has rather limited value for triaging patients according to the likelihood of disease (114, 118–120). On the other hand, symptoms such as hyperhidrosis, palpitations, tremor, pallor, nausea or signs such as low body mass index may be more useful in the assessment of the clinical suspicion of a PPGL (120). A detailed medical history for the detection of clinical signs and symptoms related to catecholamine excess, is therefore important before defining a PPGL as “clinically silent”.

### Non-secretory PPGLs

Although the term “clinically silent” should be used to describe the absence of signs and symptoms of catecholamine excess, the term “non-secretory” is preferably used to describe tumors that consistently show lack of catecholamine secretion as manifest by repeated samplings of blood or 24-hour urine specimens and measurements of catecholamines. Catecholamines are actively secreted from chromaffin cells or tumors, principally by a process involving exocytosis, which can occur episodically or at low rates (121). Independent of their secretion, catecholamines also leak continuously from storage vesicles into the cytoplasm of chromaffin cells. Presence of catechol-O-methyltransferase (COMT) within the cytoplasm then leads to metabolism of norepinephrine to normetanephrine and of epinephrine to metanephrine; the metabolites then diffuse passively from chromaffin cells into circulation (122, 123).

**Figure 2:**
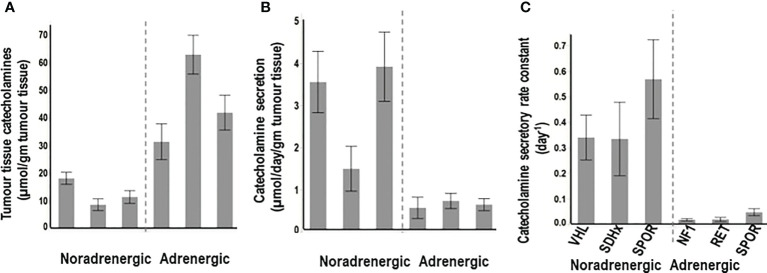
Tumor tissue contents of catecholamines **(A)**, rates of secretion of catecholamines from tumors **(B)** and catecholamine secretory rate constants **(C)** for PPGLs from patients with hereditary (*VHL, SDHx*), and sporadic (SPOR) noradrenergic tumors versus hereditary (*NF1, RET*) and sporadic (SPOR) adrenergic tumors. Secretory rate constants illustrate that for noradrenergic tumors over a third of all catecholamines in stores are secreted within one day, whereas for adrenergic tumors less than 5% of stores are secreted within one day. (*Reproduced with permission from Eisenhofer G et al. Clin Biochem Rev 2017*).

“Non-secretory” PPGLs are most often adrenergic tumors, including those due to mutations of cluster 2 genes, that despite the large amounts of tissue catecholamines (**Figure 2A**), show dense distributions of both epinephrine and norepinephrine vesicles, associated with low levels of secretory activity (**Figure 2B**). Secretion is often less than 5% of all catecholamine stores within one day (**Figure 2C**). Consequently, such tumors may present with consistently normal plasma concentrations or urinary outputs of norepinephrine and epinephrine.

Although patients with adrenergic tumors are often asymptomatic due to circulating catecholamines at entirely normal concentrations, it is important to appreciate that such “non-secretory” PPGLs remain functional with large amounts of tissue catecholamines that continuously leak into cytoplasm. There the catecholamines are metabolized to metanephrines, providing a biochemical signal more useful than the catecholamines for diagnosis (124). Also, although such tumors may be classified as non-secretory in nature, it must be appreciated that any tumor that synthesizes, stores and metabolizes catecholamines to metanephrines also has the capacity to secrete catecholamines if provoked. Indeed, the highly differentiated nature of cluster 2 tumors means that the many components of the secretory apparatus are intact and in place to limit catecholamine secretion unless a signal is received (116). The intact secretory apparatus includes receptors and secondary messenger systems that can respond to many signals including dopamine D2 receptor antagonists and glucagon (125). Provoked secretion of catecholamines from these adrenergic tumors can thereby be more easily achieved than from noradrenergic tumors or those due to mutations of pseudohypoxia genes, which secrete catecholamines more continuously than adrenergic tumors (116, 117).

One illustrative case reported as a “non-secreting” pheochromocytoma, based on consistently negative test results for urinary and plasma catecholamines, involved a woman who presented with hypertensive crises after administration of a dopamine D2 receptor antagonist (9). The patient showed complete recovery after a 2.5 cm adrenal mass was resected. Thus, simply because catecholamines may be normal, this does not imply that there is no tumor capable of secreting catecholamines if provoked. In the above case additional tests included plasma chromogranin A, urinary VMA and total metanephrines, but these are also all insensitive tests of catecholamine excess. Measurements of urinary fractionated metanephrines, or more ideally mass spectrometric measurements of plasma free metanephrines, more appropriately establish functionality, but even the latter can be negative in patients with small tumors (86).

Of course, plasma and urine catecholamines can also be consistently normal in patients with large tumors; these can include rare tumors that lack the biosynthetic machinery required for catecholamine production and that also do not produce increases in urinary fractionated or plasma free metanephrines. Although such tumors might also be labeled as “non-secretory” there are other terms as covered later that may more accurately define the nature of their biochemical and clinical presentation.

### Biochemically negative PPGLs

Review of the literature shows that patients with PPGLs and negative biochemical test results are often defined to have “biochemically silent” tumors (4, 11, 16, 18, 21, 22, 58–63). In most of the aforementioned studies, the authors define PPGLs as “biochemically silent” according to measurements of catecholamine-related biomarkers that do not exceed the upper cut-offs. Although this may be appropriate in certain circumstances, the term “biochemically silent” is often used indiscriminately. In particular, a large proportion of patients categorized with “biochemically silent” tumors simply have false-negative biochemical test results as a consequence of inappropriate choice of biochemical markers, measurement methods or even application of reference intervals. The appropriate solution is to define these tumors as “biochemically negative”. Such solutions should also clarify the particular test, analytical measurement method and associated reference intervals.

As established in the present literature review, the above information is rarely provided in manuscripts reporting on biochemically silent PPGLs. Even when some or all of the above data are supplied, there may be errors or confusion. The patient presented by Kota et al. (22) with a biochemically silent adrenal incidentaloma that resulted in an intra-operative hypertensive emergency, and was subsequently confirmed to be a pheochromocytoma, provides an illustrative example. The patient was reported to have normal pre-operative urinary fractionated and plasma metanephrines. However, review of the presented data reveals urinary metanephrines reported as a single value with reference intervals in line with spectrophotometric measurements of total metanephrines rather than contemporary measurements of the fractionated metabolites. Even more strikingly, plasma measurements were similarly reported as a single value of 34 µg/dL. Though lower than the reported cut-off of 60 µL/dL, those values are more than three orders of magnitude higher than established plasma concentrations and also well beyond the range of either normetanephrine or metanephrine for patients with pheochromocytoma. Such reports are emblematic of a general lack of clinical understanding of measurement methods and biochemical tests.

Despite recommendations of the Endocrine Society clinical practice guidelines that biochemical diagnosis of PPGLs should be based on measurements of plasma free or urinary fractionated metanephrines (2) many clinicians still rely on measurements of catecholamines, vanillylmandelic acid (VMA) and/or chromogranin A (CgA) for diagnosis of PPGLs. As covered earlier, metanephrines are produced within chromaffin cells by COMT, an enzyme absent in sympathetic nerves. This means that the O-methylated metabolites are much more specific for chromaffin cells and PPGLs than their parent catecholamines or any other catecholamine metabolites. Consequently, about 8-9% of patients with sporadic PPGLs and 21-31% with hereditary PPGLs, have normal plasma concentrations and/or urinary outputs of catecholamines but show elevations of plasma metanephrines (126). Apart from the importance of measuring metanephrines rather than catecholamines, the benefits of additional measurements of methoxytyramine in plasma should also be considered. This assists not only with confirmation of disease but also with detection of predominantly dopamine producing tumors (108, 127).

Although the superiority of metanephrines over catecholamines is clear, superiority of measurements in plasma over urine was only recently clearly established. In particular, Eisenhofer and colleagues (86), showed that urinary fractionated metanephrines and methoxytyramine have a significantly lower sensitivity (92.9%) compared to plasma free metanephrines and methoxytyramine (97.9%). The above findings can be explained by the large amounts of normetanephrine and dopamine formed in the body that are produced and metabolized within mesenteric organs (128). This confuses the diagnostic signal of urinary normetanephrine and methoxytyramine, which are commonly measured in urine after acid hydrolysis catalyzed deconjugation of sulfate conjugated metabolites to free metabolites. The sulfate-conjugated metabolites are the main species present in urine and their synthesis from the actions of a specific sulfotransferase isoenzyme, SULT1A3, localized to gastrointestinal tissues, acts to dilute the signal of the free metabolites produced elsewhere in the body including in chromaffin cell tumors. The additional substantial impact of dietary derived dopamine on sulfate conjugated metabolites of dopamine and its metabolite methoxytyramine further reduces any diagnostic signal for urinary methoxytyramine measured after acid hydrolysis (129).

Apart from the appropriate choice of biochemical markers, appropriate choice of measurement methods is also crucial for the accurate diagnosis or exclusion of PPGLs. Among analytical methods, liquid chromatography with electrochemical detection (LC-ECD) (122), and liquid chromatography with tandem mass spectrometry (LC-MS/MS) (130) offer superior diagnostic performance compared to immunoassays (131). Although LC-ECD is well established for measurements of urinary fractionated metanephrines, many clinicians continue to rely on immunoassay measurements of plasma metanephrines, which is associated with false negative results in up to a quarter of all patients with PPGL (131). The significant drop in the diagnostic sensitivity is explained by problems with calibration, in particular lack of commercially available L-isomers, which resulted in measurements that are 60% lower than true concentrations. The problem is further compounded by use of inappropriately high upper cut-offs of reference intervals.

Inappropriately high upper cut-offs can also be a problem for other methods used for measurements of plasma free metanephrines. In particular, some laboratories have set cut-offs of reference intervals determined from blood samples obtained from patients in the seated position, which results in an activated sympathetic nervous system and increased plasma concentrations of norepinephrine and normetanephrine. The associated reference intervals are too high for reliable confirmation of PPGL, as well as exclusion of PPGL, which as recommended by Endocrine Society guidelines should be established from samples taken in the supine position.

Although most of the “biochemically silent” PPGLs described in the literature probably involve cases with false negative test results, there are occasional patients with truly “biochemically silent” PPGLs. Apart from the non-functional tumors that are described in detail below, functional PPGLs of small size (usually <1 cm) at an early stage of development may present with negative biochemical test results. Indeed, surveillance programs and widespread use of imaging techniques have led to the increased detection of such small PPGLs, which despite their functionality are still too small to produce sufficient amounts of catecholamines and therefore meaningful increases in plasma or urinary catecholamines and their metabolites. This can be easily understood when the strong association between tumor size and the extent of increases in summed plasma concentrations of metanephrines is considered (89). As mentioned above, this association is based on the continuous production of metanephrines within the tumor cell cytoplasm, which depends on passive leakage of catecholamines from vesicular stores, the size of which relate to tumor burden (123, 124).

### Non-functional PPGLs

Correct determination of functionality – in terms of whether PPGL synthesize, store and have potential to secrete catecholamines – can be important in determining need for pre-operative α-adrenoceptor blockade to avoid potential danger of catecholamine hypersecretion that might be provoked during surgical intervention. Even a small PPGL or those associated with normal biochemical test results can produce dangerous increases in blood pressure (132–134). Hypertensive crises during adrenalectomy have been reported in patients with negative biochemical test results (8) including one case involving development of pulmonary edema that required a seven day intensive care unit recovery (4). Increasingly inappropriate use of the term, “non-functional”, may be misleading to some who may incorrectly determine lack of need for α-adrenoceptor blockade.

“Non-functional” PPGLs, are tumors that neither synthesize nor secrete catecholamines, often located in the head and neck (HNPGL) or rarely the upper/anterior mediastinum (135). Only 3-4% of HNPGLs produce norepinephrine (136), though as much as 1/3 of all HNPGLs may produce some dopamine (137). In cases of total absence of catecholamine production, HNPGLs can be defined as “non-functional”. Abdominal “non-functional” PPGLs are extremely rare, but when found may be due to *SDHB* mutations (58). Lack of catecholamine secretion and metabolism by these tumors may result from a defect in the synthesis of catecholamines due to absence of tyrosine hydroxylase, rather than a defect in the storage or release of catecholamines.

Tyrosine hydroxylase is responsible for conversion of L-dopa to dopamine and represents the rate limiting enzyme in catecholamine synthesis (138). Thus, absence of this or other critical enzymes, such as dopamine beta-hydroxylase (139), is expected to lead to absence of tumor tissue catecholamines and a “non-functional” presentation (**Table 3**). Such tumors, similar to those that produce predominantly dopamine, tend to reach a large size before diagnosis, which is usually due to local mass effects and incidental discovery on imaging. Biochemically negative PPGL that are characterized by an immature biochemical phenotype and low tissue stores of catecholamines, only some of which may be truly “non-functional” (58), are associated with an aggressive phenotype in terms of higher rates of malignancy (140–144).

**Table 3:** Expected biochemical test results and features in biochemically negative, non-secretory and non-functional tumors.

Tumor type	Plasma or urinary catecholamines	Plasma or urinary metanephrines	Catecholamine synthesizing enzymes	Tumor tissue catecholamines
Biochemically negative	Normal	Normal	Present if functional	Present if functional
Non-secretory	Normal	Elevated	Present	Present
Non-functional	Normal	Normal*	Absent/undetectable	Undetectable/low†

*If tumor tissue catecholamines and/or catecholamine synthesizing enzymes cannot be assessed, then a non-functional tumor may be defined by plasma or urinary metaneprhrines that are too low according to relationships with tumor size. ^†^Catecholamines are potentially measureable in any human tissue, but for a tumor that is non-functional tissue catecholamines are considerably lower than in functional PPGLs.

With the above considerations in mind, the definitive method to establish absence of functionality in a PPGL is through measurements of tumor tissue catecholamines as illustrated by Timmers et al. (58). Additional measurements of tumor tissue tyrosine hydrolase activity can also be useful, as can be immunohistochemical analyses for the presence of catecholamine-synthesizing enzymes (145, 146). However, immunohistochemical presence of enzymes involved in catecholamine synthesis does not always translate to functional synthesis and storage of catecholamines in secretory granules (121). Lack of secretory granules with electron microscopy can also point to a non-functional paraganglioma. However, presence of secretory granules may not necessarily indicate a tumor with functional capacity to synthesize and store catecholamines, since the electron dense nature of such granules reflects presence of chromogranins and it can be possible for granins to be present in secretory granules without presence of catecholamines (58). Also, as reported in two studies (147, 148), since dopamine is produced in the cytoplasm while production of norepinephrine requires translocation of dopamine into secretory granules, lack of secretory granules but presence of tyrosine hydroxylase might be responsible for some cases of exclusively dopamine-producing tumors. This may also be the situation in HNPGLs that produce methoxytyramine from dopamine (149).

**Figure 3:**
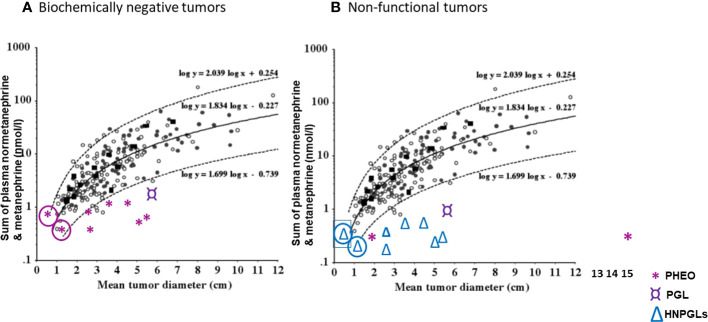
**(A)** Correlation of original tumor size with the sum of plasma normetanephrine and metanephrine (nmol/l) in seven patients (*) with biochemically negative PPGLs from the study of Gruber et al. (64). **(B)** Correlation of original tumor size with the sum of plasma normetanephrine and metanephrine (nmol/l) in seven patients (△) with head and neck paraganglioma (HNPGLs), one (¤) with abdominal paraganglioma (PGL) and two with pheochromocytomas (PHEO), from the study of Eisenhofer et al. (89) and Heavner et al. (90). In circle are shown two patients with questionable tumor functionality. (*Reproduced with permission from Eisenhofer G et al. Clin Chem 2015*).

Pre-operatively, lack of functionality may be suspected through considerations of tumor size and plasma metanephrines (89). Since the sum of plasma free metanephrines is positively related to tumor size it can be possible to identify which tumors are likely to be non-functional rather than simply biochemically negative (**Figure 3**). For instance, in the study of Gruber et al. (64), the authors defined seven patients with pheochromocytomas and biochemical negative results as biochemically “silent”. On inspection of the relationship of tumor size with the sum of plasma metanephrines (**Figure 3A**), it could be determined that for all seven patients the sum of plasma metanephrines falls within the expected relationship with tumor size, indicating that the negative biochemical signal for the tumors in those patients most likely reflected their small size rather than any lack of functional production of catecholamines. In other words, tumor size was small and the associated total catecholamine contents were unlikely sufficient to produce a positive biochemical signal.

In the report that assessed functionality according to tumor size in relation to plasma metanephrines (89), one of 207 patients (0.5%) with pheochromocytoma and another one of 45 patients (2.2%) with paragangliomas were defined as having non-functional tumors based on negative biochemical test results and a mean tumor diameter of larger than 2 cm. This compared to 12 of 43 patients (28%) with HNPGL defined to have non-functional tumors by the same criteria. Other patients with negative biochemical test results and mean diameters less than 2 cm, including 11 of the 43 patients with HNPGL (26%), were defined as having indeterminate catecholamine biochemical phenotypes. Thus, in those patients as well as 3 of 207 patients with pheochromocytoma who had negative biochemistry, functionality could not be excluded. In another report by Heavner et al. (90) in which seven pheochromocytomas were appropriately defined as biomarker negative, there were two patients reported with biochemically negative results for plasma metanephrines, one with a 1.7 cm tumor and the other with a 15 cm tumor. The latter large tumor could therefore be defined as non-functional, while for the former 1.5 cm tumor lack of function could not be determined. There was another patient with a 6.3 cm tumor in whom tests of plasma free metanephrines were indicated as normal, though not reported. That patient most likely also had a non-functional tumor. For the other four cases, either biochemical tests were inadequate or tumors were too small to determine functionality.

As illustrated in **Figure 3B**, a selection of cases from the above two reports (89, 90) serves to clarify situations, other than those that verify absence of tyrosine hydroxylase and tumor tissue catecholamines, where the term “non-functional” might be applied to patients with PPGL who present with negative biochemical test results for plasma free metanephrines.

Relationships of tumor size with urinary metanephrines have yet to be adequately determined. Therefore, determinations of non-functional versus functional status are more difficult for urinary than plasma measurements. Also, among 236 patients with PPGLs in a previous report (86), 16 patients had negative test results for urinary fractionated metanephrines compared to 5 with negative results for plasma free metanephrines. Thus, negative test results for measurements of urinary metanephrines more usually do not indicate a non-functional tumor, but rather reflect relative lack of diagnostic sensitivity. Moreover, in that study two of the five patients with previously negative results for plasma free metanephrines showed positive test results after three to six years of further testing when tumors enlarged.

For plasma or urinary catecholamines such determinations of functionality from relationships with tumor size are not possible. Thus, for these and most other situations involving biochemical test results that fall below upper cut-offs of reference intervals, rather than defining the tumors as non-functional or non-secretory, it is more appropriate to indicate the tumors as biochemically negative.

## Summary of proposed nomenclature

**Figure 4:**
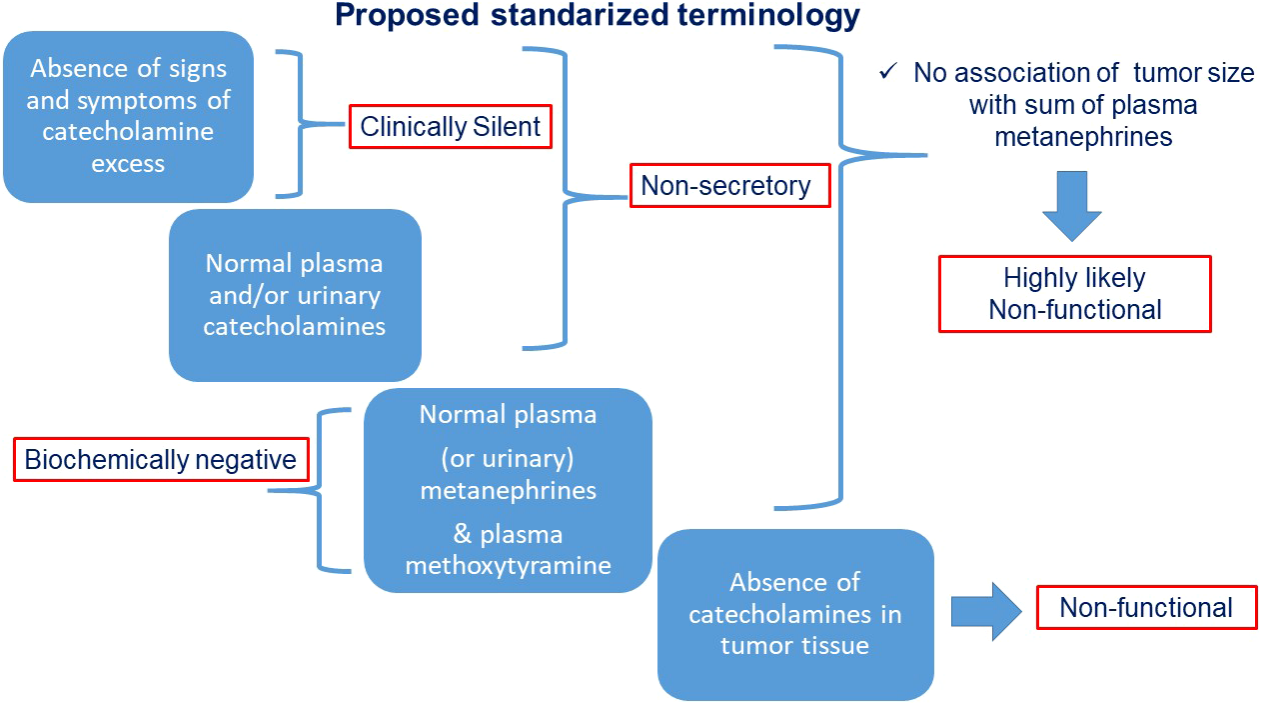
Chart flow with proposed standardized terminology for various types of “silent” PPGLs.

To facilitate scientific communication and consistent interpretation, we propose definitions for the various types of “silent” PPGLs as illustrated in **Figure 4** and outlined below.

- “Clinically silent” PPGLs are those characterized by the absence of signs and symptoms associated with catecholamine excess.
- “Non-secretory” tumors are those with absence of clear catecholamine secretory activity, often adrenergic and presenting with normal plasma and/or urinary catecholamines over multiple sampling time points.
- “Biochemically negative PPGLs are those characterized by plasma or urinary metanephrines below the upper cut-offs of reference intervals. If only catecholamines are measured the same term may be used with clarification
- “Non-functional” tumors are those with absent catecholamine synthesis as determined from measurements of catecholamines in the tumor tissue, assessments of tumor tissue tyrosine hydroxylase or large size in association with negative results for plasma or urinary metanephrines.

The above aspects are important to consider in daily clinical practice for individualized management and treatment of patients with PPGLs. In particular, “clinically silent” and “non-secretory” tumors are usually functional, and pre-surgical treatment with α-adrenoceptor blockade is essential to minimize intraoperative hemodynamic instability. In patients presenting with negative biochemical test result, the reliability of measurements should be verified. A negative biochemical test result cannot alone exclude functionality, especially for smaller PPGLs (<2 cm). Unless, functionality is correctly excluded, pre-operative blockade of adrenoreceptors remains important.

## Data availability statement

The original contributions presented in the study are included in the article/supplementary material. Further inquiries can be directed to the corresponding authors.

## Author contributions

Conceptualization, GC and CP. Methodology, VC, TS. data curation, CPr, TS. Writing—original draft preparation, GC, VC, CP. Writing—review and editing GC, GE, JL, CP. Supervision, GE, JL, CP, SB. All authors contributed to the article and approved the submitted version.

## Funding

This work was supported by the Deutsche Forschungsgemeinschaft (CRC/Transregio 205/2; to GC, SB, JL, GE, CP).

## Acknowledgments

This work is part of a Master’s thesis of the Master’s Program in Clinical Research, Dresden International University, Dresden, Germany.

## Conflict of interest

The authors declare that the research was conducted in the absence of any commercial or financial relationships that could be construed as a potential conflict of interest.

## Publisher’s note

All claims expressed in this article are solely those of the authors and do not necessarily represent those of their affiliated organizations, or those of the publisher, the editors and the reviewers. Any product that may be evaluated in this article, or claim that may be made by its manufacturer, is not guaranteed or endorsed by the publisher.
